# Supplementary material for: Avian Diversity Responds Unimodally to Natural Landcover: Implications for Conservation Management
Source: Animals (Basel). 2023 Aug 16;13(16):2647. doi: 10.3390/ani13162647 (PMC10451700; doi:10.3390/ani13162647)
Supplement: Supplementary file 1 [file animals-13-02647-s001.zip › animals-2505405-supplementary.pdf]

[supplementary material]

Article

Avian Diversity Responds Unimodally to Natural Landcover: Implications for Conservation Management

Table S1. Results of the MOStest [48] applied for avian richness-landcover relationships in both study areas (ON and NY State) and across different grain sizes. The test's null hypothesis is that richness humps of a quadratic function of the proportion of natural landcover (pNLC) at its min or max.

Grain size / Study area	Humps of a quadratic function of pNLC		F-stats	p-values
	Min	Max		
**5x5km**				
NY State	Hump min at	0.63	685	P<0.0001
	Hump max at	100.00	918	P<0.0001
**10x10km**				
NY State	Hump min at	4.67	236	P<0.0001
	Hump max at	100.00	276	P<0.0001
Ontario	Hump min at	0.18	390	P<0.0001
	Hump max at	99.00	440	P<0.0001
**30x30km**				
NY State	Hump min at	0.90	59	P<0.0001
	Hump max at	99.00	30	P<0.0001
Ontario	Hump min at	9.70	24.40	P<0.0001
	Hump max at	97.20	49.50	P<0.0001

Table S2. OLS model results for predictors of total avian richness in landscapes of three different sizes in southern Ontario (ON) and New York State (NYS) [equivalent to Table 2 – Main text, without spatial correlation]. Predictors included in full models: Annual Mean Temperature (MAT), the Proportion of Natural Land Cover (pNLC), and Land Cover Variety (LVC), and covariates: sampling effort (E - Ontario data only) and the size (richness) of regional pool of species (Pool). n represents the number of landscapes in each study area. AICc is the Akaike's information criterion corrected for sample sizes [59]. Adj.-R^2 is the adjusted model goodness of fit. Non-statistically significant terms (p>0.05) showed in brackets.

Landscape sizes	Study area	Standardized coefficients										AICc	Adj.R^2
		MAT	MAT2	pNLC	pNLC2	LCV	LCV2	E	E2	Pool	Pool2		
5x5km	NYS (n=4,822)	2.12	-2.11	1.18	-1.20	0.20	(0.13)	0.25	-0.42	0.91	-0.75	12184	0.27
10x10km	NYS (n=1,075)	2.53	-2.53	1.41	-1.36	0.60	-0.53	0.31	-0.50	(-0.04)	(0.20)	2646	0.32
	ON (n=985)	1.56	-1.20	2.18	-1.91	0.35	0.14	0.65	-0.36	(0.66)	(-0.36)	1995	0.56
30x30km	NYS (n=165)	2.02	-2.00	1.71	-1.58	1.08	-1.13	0.45	-0.64	1.67	-1.84	419	0.31
	ON (n=138)	2.62	-2.17	1.05	-0.94	(0.29)	(-0.34)	0.82	-0.59	(-4.07)	(4.11)	258	0.65

Table S3. Autoregressive model results for predictors of **total avian richness** in landscapes of three different sizes in southern Ontario (ON) and New York State (NYS) **[equivalent to Table 2 – Main text, but models fitted with a subset of the data where 5℃≤MAT≤10℃ to reduce collinearity between temperature and landcover].** Predictors included Annual Mean Temperature (MAT), the Proportion of Natural Land Cover (pNLC), and Land Cover Variety (LVC), and covariates: sampling effort (E - Ontario data only) and regional pool of species (Pool) derived from range-maps. *n* represents the number of landscapes in each study area. AICc is the Akaike's information criterion corrected for sample sizes [59]. Nagelkerke pseudo-R^2 is the equivalent to regular adjusted model goodness of fit in OLS models. Non-statistically significant terms (p>0.05) showed in brackets.

Landscape sizes	Study Area	Standardized coefficients										AICc	Nagelkerke R^2
		MAT	MAT^2	LC	LC^2	LCV	LCV^2	E	E^2	Pool	$Pool^2$		
5x5km	NYS (n=4094)	1.86	-1.85	1.17	-1.14	0.38	-0.32	0.26	-0.36	3.90	-3.78	11045	0.14
10x10km	NYS (n=932)	2.16	-2.77	-0.45	-0.74	(0.26)	(-0.25)	0.35	-0.25	(2.18)	(-2.09)	2419	0.24
	ON (n=654)	1.15	-1.02	1.46	-1.12	0.41	(-0.05)	0.68	-0.38	2.34	(-2.33)	1213	0.63
30x30km	NYS (n=139)	2.70	-2.82	1.82	-1.80	1.66	(-1.70)	0.44	-0.54	(3.62)	(-3.80)	339	0.42
	ON (n=81)	1.70	-1.92	1.65	-1.30	(0.60)	(-0.65)	0.85	-0.56	(3.80)	(-3.40)	154	0.70

Figures (1-8)

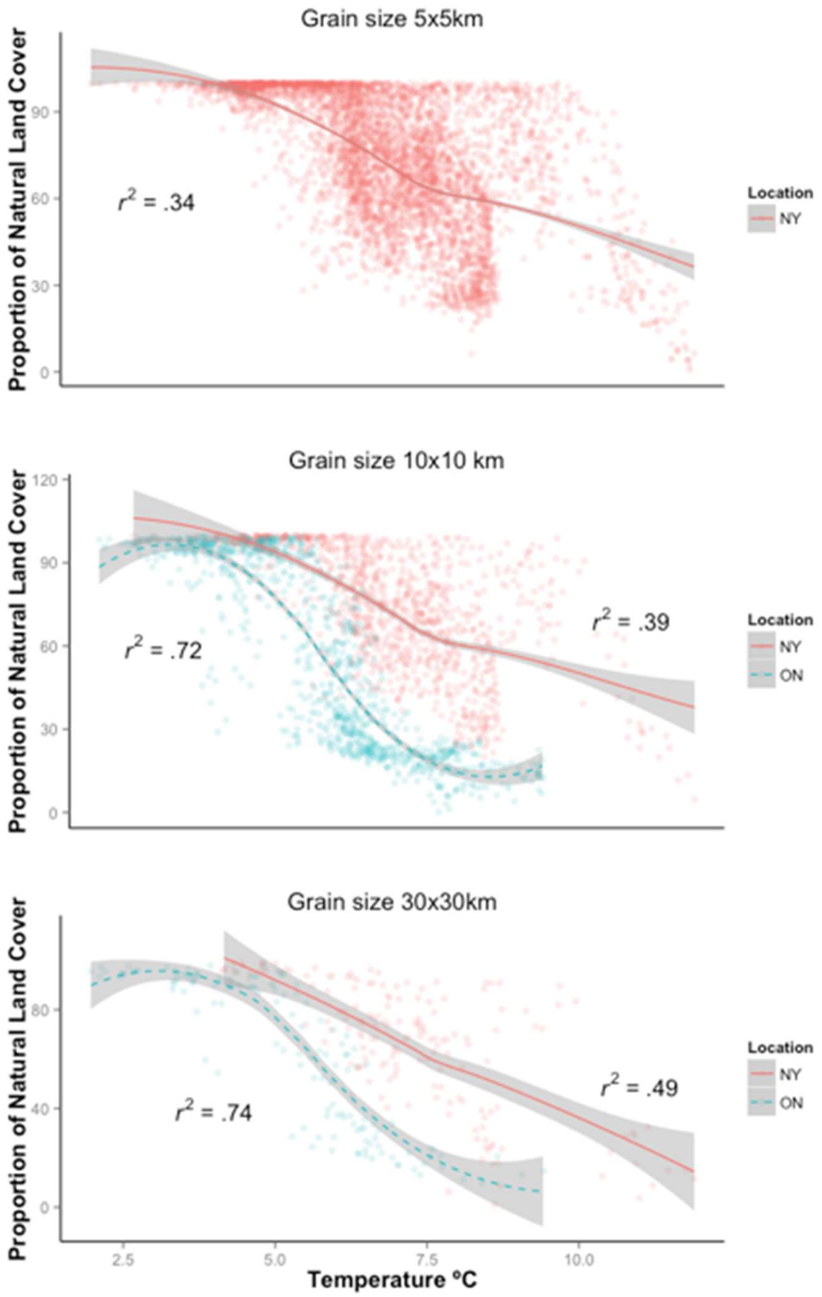

Figure S1. Linear regressions between the proportion of natural landcover and temperature at different spatial grain sizes. r^2 represents the goodness of fit of models (r-squared) fitted between the two variables.

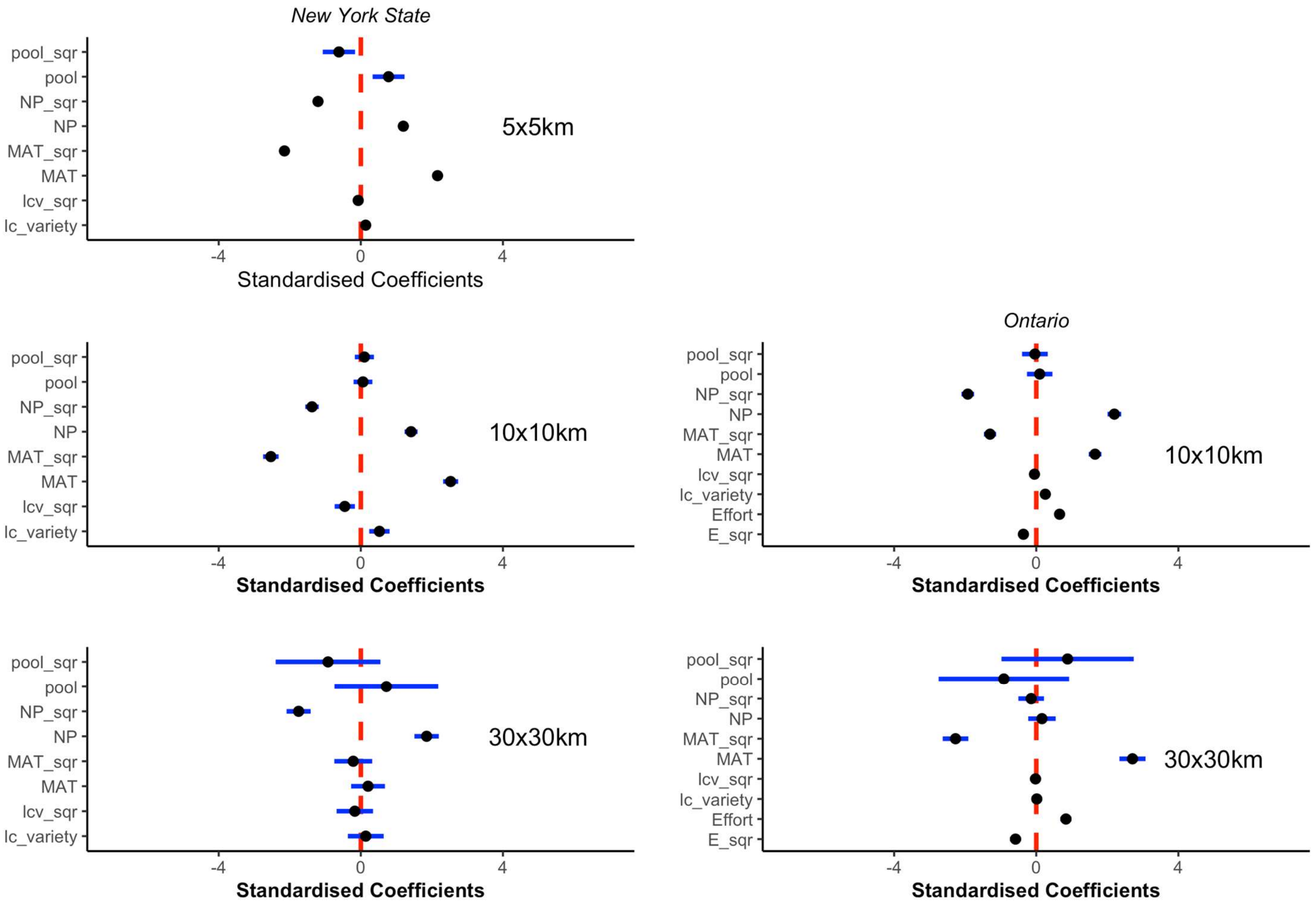

Figure S2. Multiple regressions coefficients of the predictors (annual mean temperature - MAT, proportion of natural landcover - NP, landcover variety - lc_variety), covariates (regional pool richness – pool, and sampling effort), as well as predictor squared terms on **total species richness derived from atlas data**, in 3 different grain sizes and two regions southern Ontario (left panel) and New York State (right panel). Points are the standardized, averaged coefficients (weighted by the model's Akaike weight) from all models in a 95% confidence set and error bars are the associated unconditional confidence limits.

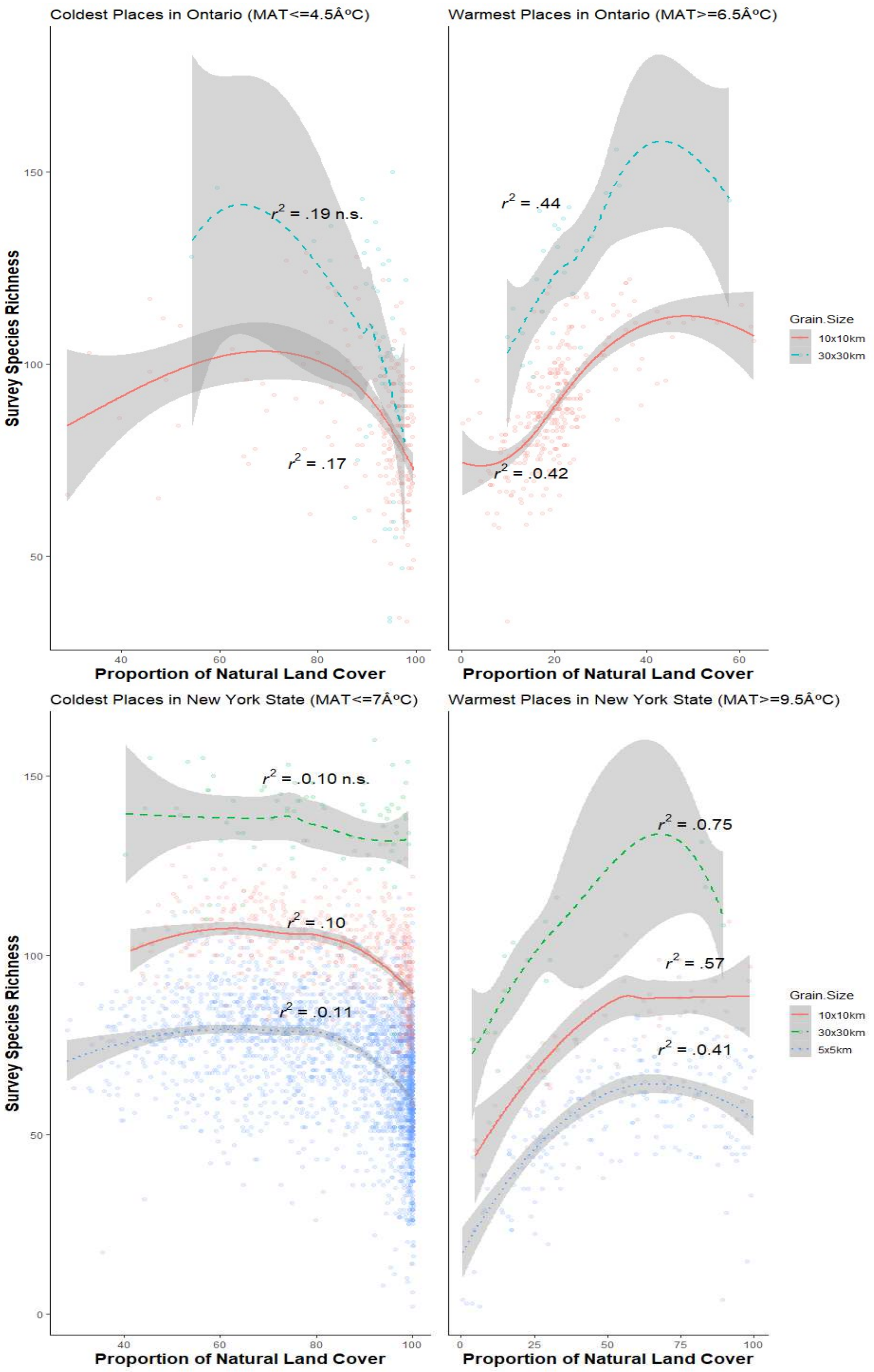

Figure S3. Survey richness (species richness derived from Altas) shows a relatively peaked shape curve when regressed against natural landcover relationships in the warmest and coldest places in southern Ontario and New York State. a) Coldest places in New York State (5x5km – n=1,097, 10x10km – n=244, 30x30km – n=28 cells); b) Warmest places in New York (5x5km – n=240, 10x10km – n=40, 30x30km – n=15 cells); c) Coldest places in Ontario (10x10km – n=243, 30x30km – n=46 cells); d) Warmest places in Ontario (10x10km – n=291, 30x30km – n=26 cells). *r2* represents the goodness of fit of models (r-squared) fitted between the two variables.

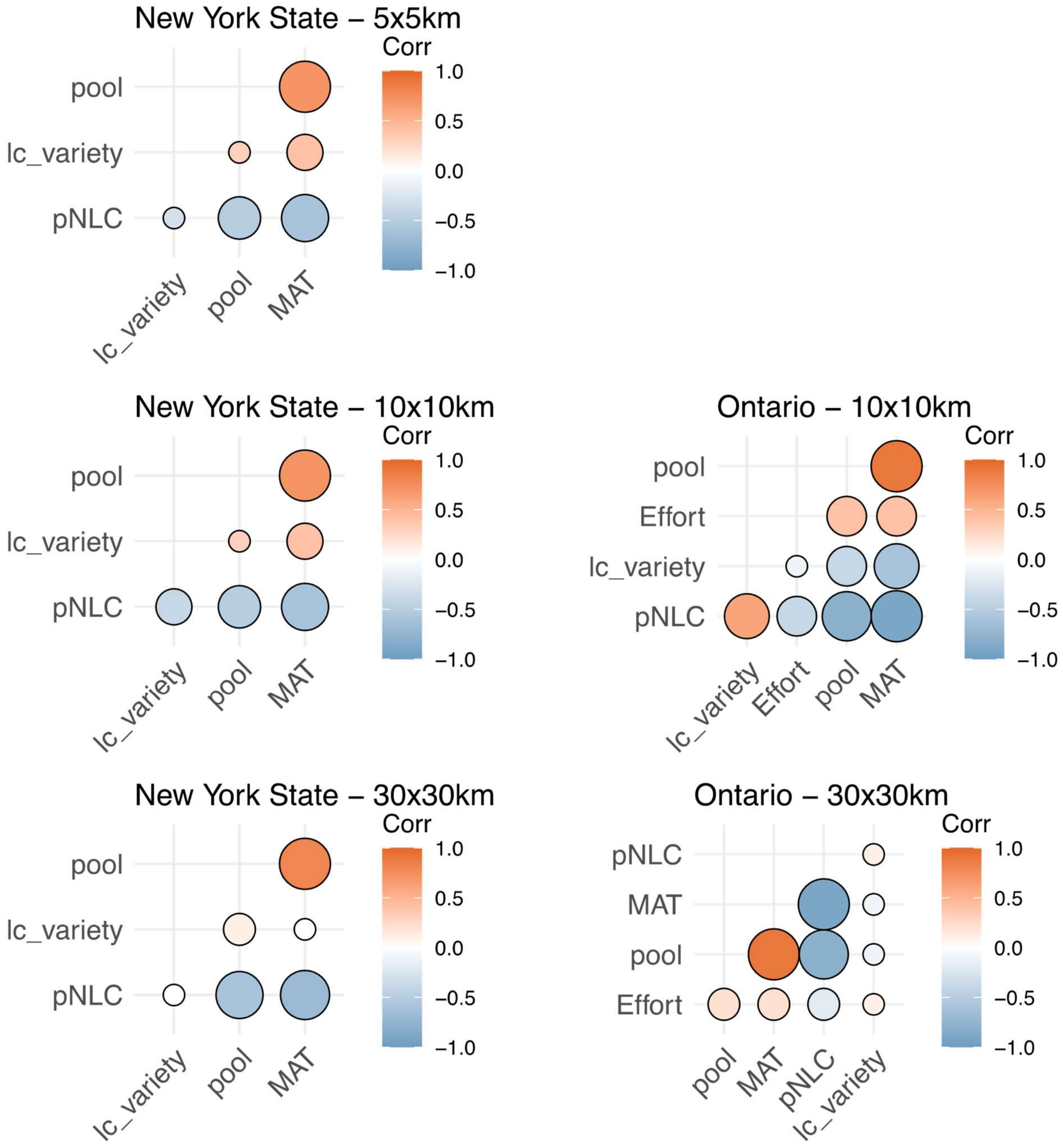

Figure S4. Pearson's correlations between independent variables used in multiple regressive models. pNLC – the proportion of natural landcover; MAT – the annual meant temperature; lc_variety - the variety of landcover types; Effort – sampling effort for Ontario data only, and Pool – regional pool of species. Variables extracted for both Ontario and New York State and across different grain sizes (5x5km, 10x10km and 30x30km).

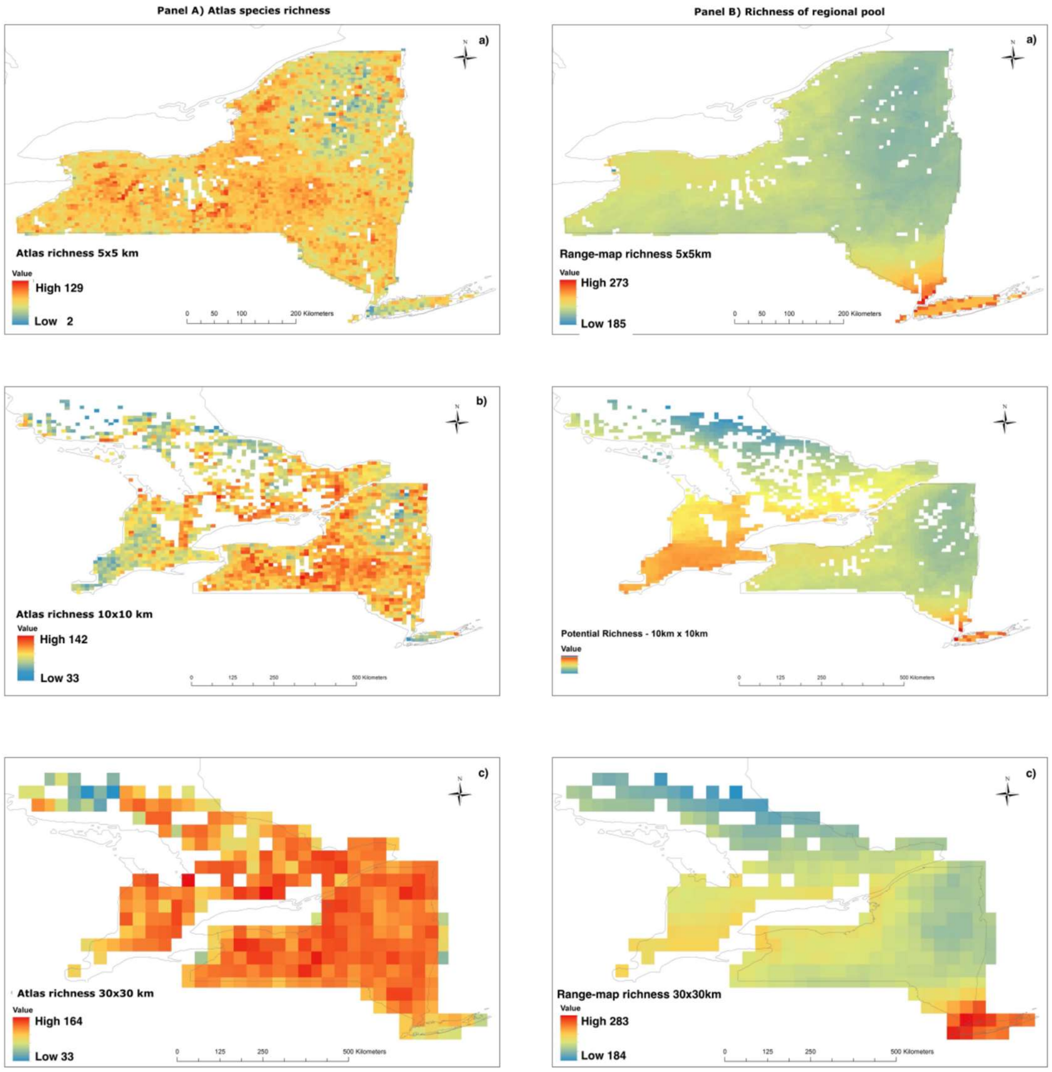

Figure S5. Panel A) Distribution of atlas species richness in a) 4,822 cells of 25-km^2 in NY, b) 2,060 cells of 100-km^2 in ON and NY, and c) 303 cells of 900-km^2 in ON and NY. Panel B) Distribution of range-map avian species richness (i.e., the species pool) in a) 4,822 cells of 25-km^2 in NY, b) 2,060 cells of 100-km^2 in ON and NY, and c) 303 cells of 900-km^2 in ON and NY. The projection is WGS84 datum.

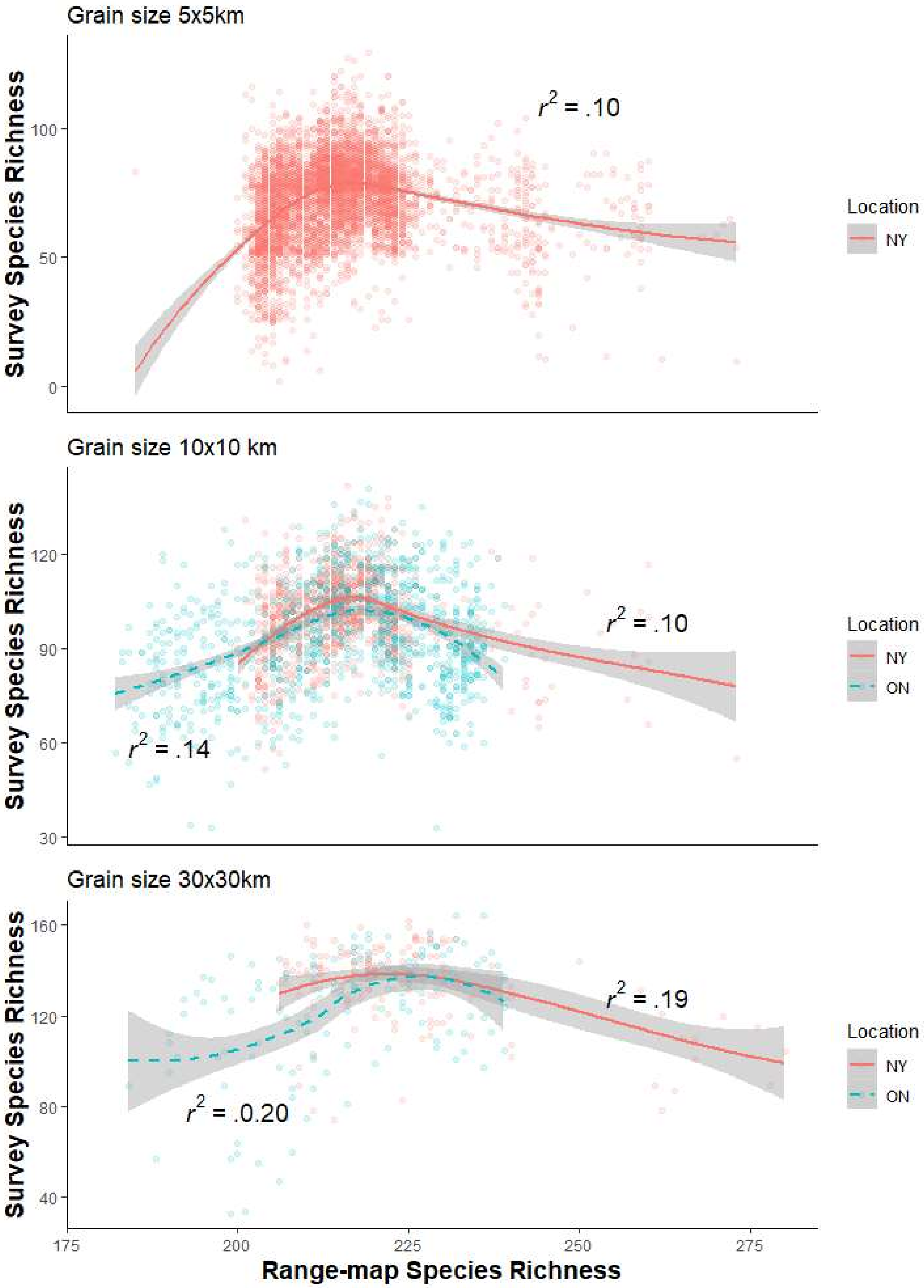

Figure S6. Survey richness (richness derived from Atlas surveys) as a function of regional pool of species (Range-maps species richness) of at different spatial grain sizes. a) n=4,822 in NY (5x5km), b) 985 and 1,075 for ON and NY, respectively, at 10x10km scale, and 251 covering ON and NY (30x30 km). *r2* represents the goodness of fit of models (r-squared) fitted between the two variables.

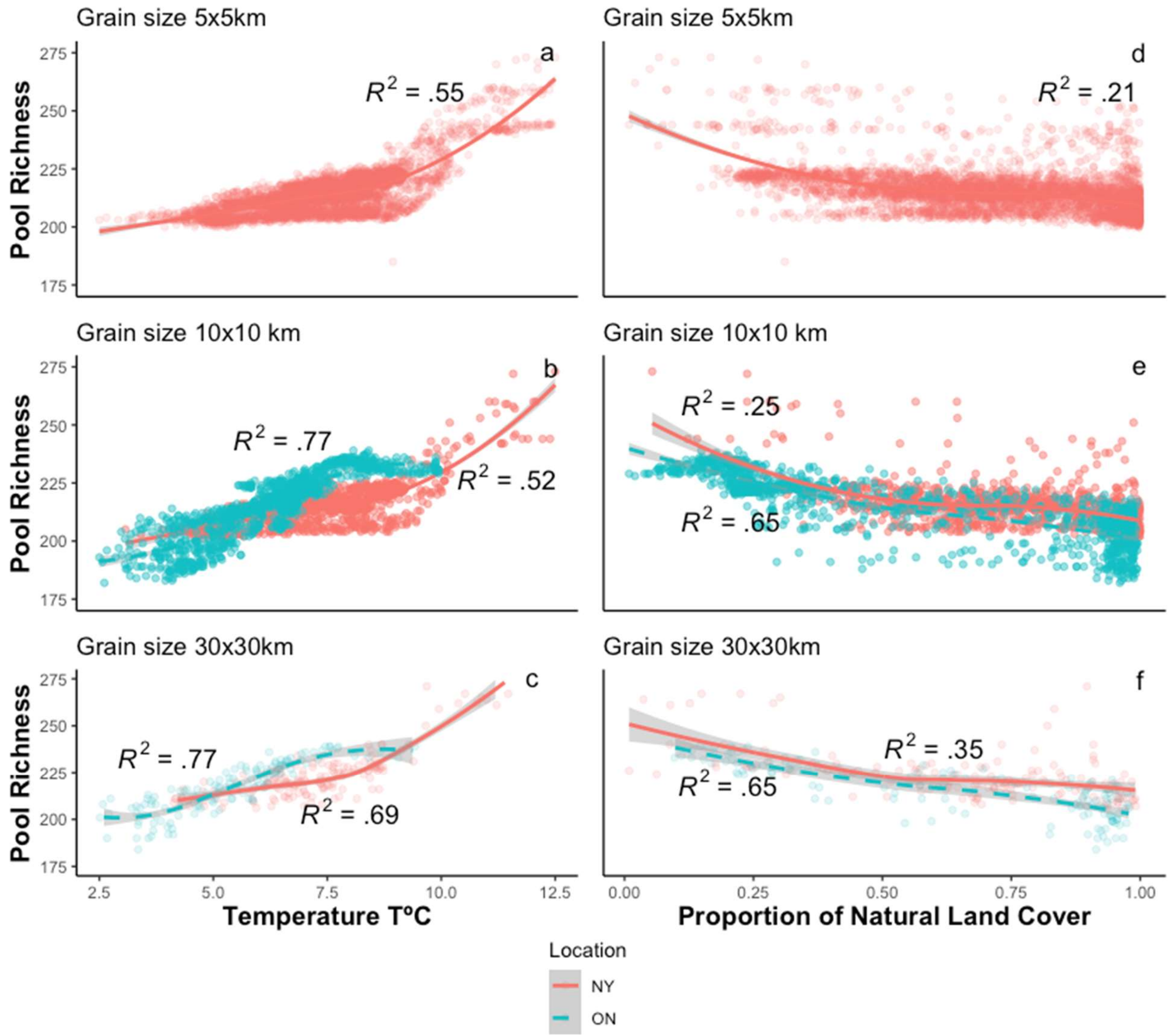

Figure S7. Relationships of **range-map richness** (the species pool) with both temperature (a, b, c) and landcover (e, e, f) in grid cells covering southern Ontario and New York State at different spatial grain sizes (5x5km, 10x10km and 30x30km). *r2* represents the goodness of fit of models (r-squared) fitted between the two variables.

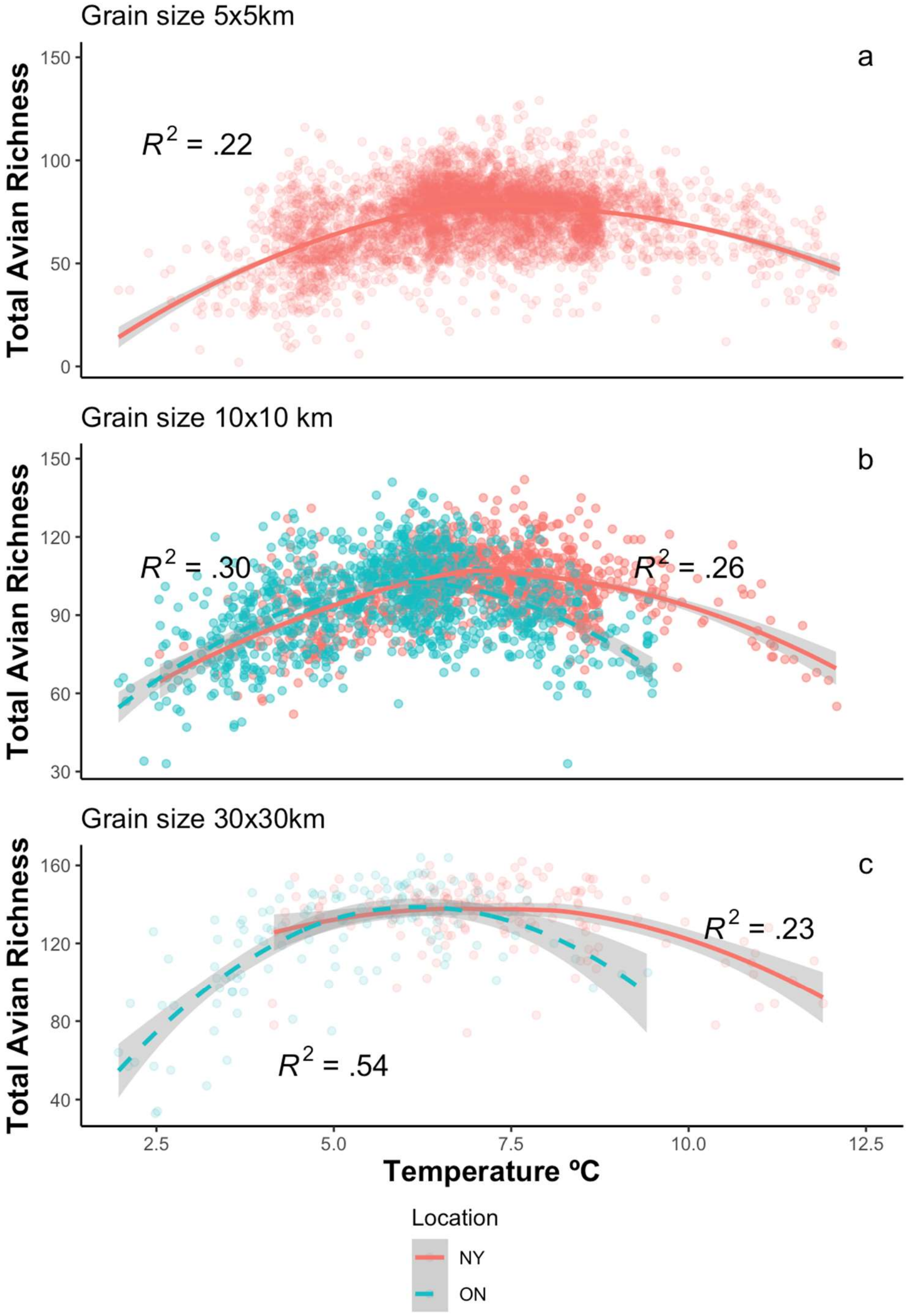

Figure S8. Relationships between temperature total avian species richness and temperature in landscapes covering southern Ontario and New York State at different spatial grain sizes (5x5km, 10x10km and 30x30km). r^2 represents the goodness of fit of second-degree polynomial OLS regression models. Panel A) Survey richness peaks at 62% natural cover in 5x5km quadrats in NY (n=4,822), 64% in 10x10km in NY (n=1,075), 64% in 30x30km quadrats in NY (n=165), 54% in 10x10km quadrats in ON (985), and 50% in 30x30km quadrats in ON (n=138). *r2* represents the goodness of fit of models (r-squared) fitted between the two variables.
